# Cannabidiol and the Canonical WNT/β-Catenin Pathway in Glaucoma

**DOI:** 10.3390/ijms22073798

**Published:** 2021-04-06

**Authors:** Alexandre Vallée, Yves Lecarpentier, Jean-Noël Vallée

**Affiliations:** 1 Department of Clinical Research and Innovation (DRCI), Foch Hospital, 92150 Suresnes, France; 2 Centre de Recherche Clinique, Grand Hôpital de l’Est Francilien (GHEF), 6-8 rue Saint-Fiacre, 77100 Meaux, France; yves.c.lecarpentier@gmail.com; 3 Centre Hospitalier Universitaire (CHU) Amiens Picardie, Université Picardie Jules Verne (UPJV), 80054 Amiens, France; valleejn@gmail.com; 4 Laboratoire de Mathématiques et Applications (LMA), UMR CNRS 7348, Université de Poitiers, 86000 Poitiers, France

**Keywords:** WNT/β-catenin pathway, cannabidiol, inflammation, oxidative stress, glutamatergic pathway, glaucoma

## Abstract

Glaucoma is a progressive neurodegenerative disease which constitutes the main frequent cause of irreversible blindness. Recent findings have shown that oxidative stress, inflammation and glutamatergic pathway play key roles in the causes of glaucoma. Recent studies have shown a down regulation of the WNT/β-catenin pathway in glaucoma, associated with overactivation of the GSK-3β signaling. WNT/β-catenin pathway is mainly associated with oxidative stress, inflammation and glutamatergic pathway. Cannabidiol (CBD) is a non-psychotomimetic phytocannabinoid derived from Cannabis sativa plant which possesses many therapeutic properties across a range of neuropsychiatric disorders. Since few years, CBD presents an increased interest as a possible drug in anxiolytic disorders. CBD administration is associated with increase of the WNT/β-catenin pathway and decrease of the GSK-3β activity. CBD has a lower affinity for CB1 but can act through other signaling in glaucoma, including the WNT/β-catenin pathway. CBD downregulates GSK3-β activity, an inhibitor of WNT/β-catenin pathway. Moreover, CBD was reported to suppress pro-inflammatory signaling and neuroinflammation, oxidative stress and glutamatergic pathway. Thus, this review focuses on the potential effects of cannabidiol, as a potential therapeutic strategy, on glaucoma and some of the presumed mechanisms by which this phytocannabinoid provides its possible benefit properties through the WNT/β-catenin pathway.

## 1. Introduction

Glaucoma is a progressive neurodegenerative disease that constitutes the main frequent cause of irreversible blindness. The number of people with glaucoma worldwide will increase from 76.5 million in 2020 to 111.8 million by 2040, mainly due to the aging of the population [1,2,3]. Glaucoma is characterized by loss of retinal ganglion cells (RGCs), thinning of the retinal nerve fiber layer, and cupping of the optic disc [4]. Glaucoma is a group of heterogeneous diseases characterized by varying clinical features. Aging, increased intraocular pressure (IOP), and genetic background are the main risk factors for glaucoma [4]. Primary open-angle glaucoma (POAG) is the main form in Western countries. Nevertheless, 30% of Caucasian patients with POAG, and a greater proportion of the Asian population show normal-tension glaucoma (NTG) [5]. The etiology of POAG is mainly described as mechanical and/or vascular processes. The mechanical process enhances compression of the axons due to elevation of IOP, whereas the vascular process highlights events in which blood flow and ocular perfusion pressure are diminished in the posterior pole [6,7]. Vascular or perfusion dysregulations in NTG present different clinical features, such as migraine headaches, Raynaud’s phenomenon or sleep apnea [8]. In high IOP glaucoma, both the anterior and posterior segments are damaged, and extensive affection is detectable in the trabecular meshwork (TM) and along the inner retina-central visual pathway [9].

Pathogenic processes of the neurodegenerative mechanism lead to mechanical and vascular stress enhancing mitochondrial dysregulation, chronic oxidative stress (OS) and metabolic stress [10,11], excitotoxicity [12], and neuroinflammation [13,14]. OS and cell senescence are increased in the aging retina [15,16] and are considered as the major glaucoma risk factors. In the aging retina, OS leads to the activation of a local para-inflammation of various magnitudes [17]. Para-inflammation, in glaucoma, is characterized by a tissue adaptive response to noxious stress [17]. However, a physiological stage of para-inflammation is needed to maintain homeostasis but when tissue is exposed to chronic stress, inflammation may have a negative role and could be involved in both initiation and progression of the disease [18]. The deregulation of para-inflammation, in the retina, is a response to stress stimuli, especially chronic OS. However, excessive and uncontrolled para-inflammation could implicate inflammatory responses with a release of cytokines/chemokines leading to neuroretina damages [19]. Para-inflammatory dysregulation could be associated with TM dysfunction and increased resistance to aqueous outflow, the main cause of increased IOP in POAG [9].

The trabecular meshwork was the main pathological localization of PAOG [20]. A balance between the production and outflow of the aqueous humor can control IOP. The TM is formed by layers of trabecular beams, and is surrounded by elastic fibers, fibronectin and laminin. Abnormalities of the extracellular matrix (ECM) are involved in high IOP [21]. Recently, the WNT/β-catenin pathway has been found to be associated with the development of glaucoma in the TM [22].

Since the early 1970s, cannabinoids (CBs) have been investigated as anti-glaucoma drugs [23]. Cannabinoids are a large class of chemical components from the trichomes and the leaves of *Cannabis* plants (phytocannabinoids) or produced by pharmacological synthesis (synthetic cannabinoids). These molecules interact with cannabinoid receptor 1 (CB1) and cannabinoid receptor 2 (CB2) which are the natural receptors of endocannabinoids modulating numerous physiological mechanisms [24]. CB1 and CB2 are expressed in human retina, ciliary body and retinal pigment epithelium, administration of exogenous cannabinoids could act on numerous retinal mechanisms including signal transduction, photo-transduction and IOP control [25,26]. CBs have been widely investigated as IOP lowering treatments [27] and possess many benefits in glaucoma [25,27,28]. Besides IOP lowering capabilities, CBs present major neuroprotective actions on the nervous system [28,29]. CBs can inhibit glutamate release and diminish oxidative stress [27].

Cannabidiol (CBD) is a non-psychotomimetic phytocannabinoid derived from Cannabis sativa plant which possesses many therapeutic properties across a range of neuropsychiatric disorders [30,31]. For a few years, CBD has presented increased interest as potential anxiolytic therapy [32,33,34]. CBD has a lower affinity for CB1 but can act through other signaling ways in glaucoma. CBD downregulates GSK3-β activity, an inhibitor of WNT/β-catenin pathway [35]. Moreover, CBD has been reported to suppress pro-inflammatory signaling and neuroinflammation [36,37], oxidative stress [38] and glutamatergic pathway [39].

Thus, this review focuses on the potential effects of cannabidiol, as a potential therapeutic strategy, on glaucoma and some of the presumed mechanisms by which this phytocannabinoid provides its possible benefit properties through the WNT/β-catenin pathway.

## 2. Pathophysiology of Glaucoma

In PAOG, the IOP increase leads to the TM occlusion induced by the iris tissue [9]. Chronic contact between the iris and the TM can lead to permanent damage to the TM. The TM dysfunction and the reduction of its cellularity are the first steps to the high tension glaucoma (HTG) onset, including POAG and also PACG (primary angle-closure glaucoma). Numerous factors, including OS and aging, as well as environmental factors are implicated as the promotors of TM damage [40]. OS could be enhanced in the morphological alterations of the TM of glaucomatous eyes, due to it stimulating inflammatory response. Chronic inflammation and OS modulate each other in a vicious circle influencing cellular responses. Cultures of TM present an NF-ϰB pathway activation after exogenous stimulation including IL1 or H_2_O_2_. The NF-ϰB activation results in a significant expression of the endothelial leukocyte adhesion molecule-1 (ELAM-1), IL-1β and IL-6 [41]. ELAM-1 belongs to selectin families, which are cell adhesion molecules. The presence of ELAM-1 in POAG is considered to be a factor in the onset of TM endothelial dysfunction [42].

During glaucoma, a progressive loss of TM cells has been shown, due to the combination of both aging and stress conditions [43]. In HTG, the TM displays both chronic inflammation and tissue reprogramming mechanisms associated with OS damage and endothelial dysfunction [44]. Among the pro-inflammatory cytokines, IL6, IL1 and TNF-alpha can induce ECM remodeling and alter cytoskeletal interactions in the glaucomatous TM [42]. The alterations in the protein patterns observed in the aqueous humor (AH) of POAG patients are the consequence of the progressive loss of TM cellular integrity [45]. The TM is the most sensitive tissue of the anterior segment of the eye to oxidative stress [46]. Glaucomatous TM cells present POAG-typical molecular modifications, such as ECM accumulation, cell death, dysregulation of the cytoskeleton, advanced senescence, NF-ϰB stimulation and the release of inflammatory markers [41,47].

These findings may suggest that the IOP elevation, which occurs in glaucoma, is associated with oxidative degenerative processes damaging the human TM endothelial cells (hTMEs). Chronic exposure of TM cells to OS leads to numerous changes in the lysosomal pathway responsible for autophagia [48], as well as cell senescence with an increase in senescence-associated-galactosidase [49]. OS induces a lysosomal dysregulation and the defective proteolytic stimulation of lysosomal enzymes with a subsequent decrease in autophagic flux and the promotion of cell senescence [9].

The IOP elevation, either at the lamina cribrosa or the optic nerve head (ONH) level, leads to hypoperfusion and to reperfusion damages [50]. IOP elevation is considered as a cause of retinal ganglion cells (RGCs) damage, resulting in a retrograde transport blockade and the accumulation of neurotrophic factors at the lamina cribrosa instead of reaching the RGC soma [51]. The POAG etiology is still unclear but several risk factors have been observed as the causes of promoting its onset, such as elevated IOP, aging, gender, ethnicity, first-degree family history of glaucoma, oxidative stress, systemic and ocular vascular factors, and inflammation [52].

## 3. Oxidative Stress, Inflammation and Glutamate in Glaucoma

The mechanisms of ROS production are activated in several pathological conditions of the retina, such as glaucoma, occlusion of the central artery of the retina and age-related macular degeneration. They are enzymes, including the nicotinamide adenine dinucleotide phosphate (NADPH) oxidase, the xanthine oxidoreductase, the cytochrome P450, the mitochondrial cytochrome oxidase and the eNOS decoupled, which catalyzes the overproduction of ROS in the tissues of the vascular system [53,54]. Oxidation decreases tetrahydrobioprotein (BH4) bioavailability, whereas it increases the 7,8-dihydrobioprotein (BH2) competing with BH4 to enhance eNOS [55].

To date, the visual loss processes are not entirely elucidated in glaucoma, and ROS production plays an important role in its development [56]. ROS production rates are increased in patients with glaucoma in the aqueous humor but also in the blood serum [57]. One of the main factors for glaucoma risk is elevated IOP. A moderately elevated IOP increases ROS production levels, stimulates NOX2 expression, and endothelial dysregulation in retinal arteries, suggesting that IOP augmentation affects the vascular function of the retina [58]. However, there are other pathogenic processes linked to glaucoma, including glutamate excitotoxicity [59], which are not necessarily associated with the elevated levels of IOP [56]. It seems that the death of RGCs during a glaucoma process stimulates ROS production in vitro [60]. It has been shown that ROS production controls the immune response by stimulating the action of antigen glial cells [60]. The ROS production affects the retina, and increases IOP to induce dysfunction of the support glia, which facilitates the secondary degeneration of the RGCs in glaucoma [61].

The immune system is regulated by several inflammatory factors, such as tumor necrosis factor α (TNF-α), interleukin-6 (IL-6), vascular endothelial growth factor (VEGF) and tumor growth factor-β (TGF-β) [62]. Chronic inflammation is also responsible for the increase of cyclooxygenase 2 (COX-2, a prostaglandin-endoperoxidase synthase) [63]. Numerous cytokines (TNF-α, IL-1) activate COX-2 [64]. COX-2 stimulates ROS and RNS production [63,65]. Nuclear factor-ϰB (NF-ϰB) stimulates several pro-inflammatory factors that activate COX-2 and inducible nitric oxide synthase (iNOS) [66]. Several studies have shown that NF-ϰB stimulates the expression of TNF-α, IL-6, IL-8, STAT3, COX-2, B-cell lymphoma 2 (BCL-2), metalloproteinases (MMPs), VEGF [66], and the ROS production [67]. Furthermore, iNOS, an enzyme catalyzing nitric oxide (NO), is activated during chronic inflammation [68].

Several pieces of research have shown the mechanism by which oxidative stress can lead to chronic inflammation [69]. The imbalance caused by oxidative stress leads to damage signaling in cells [70]. The ROS production plays a central role both upstream and downstream of NF-κB and TNF-α pathways, which are the main mediators of the inflammatory response. The hydroxyl radical is the most harmful of all the ROS. A vicious loop is observed between ROS and these pathways. ROSs are generated by NADPH oxidase (NOX) system. Moreover, the modified proteins by ROS could generate an initiation of auto-immune response to stimulate TNF-α and NOX [71]. Nuclear factor erythroid-2 related factor 2 (Nrf2) is mainly associated with oxidative stress in inflammation [69]. Nrf2 is a transcription factor that binds to the antioxidant response element (ARE) [72]. Several studies have shown that Nrf2 can present an anti-inflammatory role by regulating MAPK, NF-ϰB, and PI3K pathways [73]. Thus, Nrf2 may play a major role against oxidative damages [74]. Furthermore, evidence also suggests that mitochondrial dysregulation has a significant role in the cancer mechanism [69].

Glutamate is an amino-acid responsible for the brain’s primary excitatory neurotransmission [75]. Glutamatergic neurons are embedded in every brain circuit in comparison to dopamine and serotonin which are used by a small minority of neural cells in the brain. Glutamate is the main excitatory neurotransmitter in the brain and is present in more than 50% of synapses. This signaling plays a major role in neuronal plasticity, memory and learning [76]. Rapid neurotoxicity enhanced by neuronal excitotoxin has been observed with abnormal glutamate levels [77]. In neurons, glutamate is stored in synaptic vesicles from which it is released. Glutamate release increases glutamate concentration in the synaptic cleft to bind ionotropic glutamate receptors. The main consistent candidate gene in OCD is SLC1A1 (solute carrier, family 1, member 1) gene [78]. SLC1A1 encodes for the neuronal excitatory Na+-dependent amino acid transporter 3 (EAAT3). EAAT1 and EAAT2 are the main astrocyte glutamate transporters whereas EAAT3 is the major neuronal glutamate transporter. Glutamate is converted into glutamine in astrocytes. Then, glutamine is captured by the presynaptic neurons to be re-converted into glutamate [79]. The role of the EAAT3 is to control glutamate spillover (signification de spillover?) which affects pre-synaptic N-methyl-D-asparate (NMDA) and metabotropic glutamate receptors activity [80,81]. EAAT3 activity is dysregulated by the overexpression of GSK-3β [82].

In glaucoma, the glutamate toxicity could contribute to RGC death and appears to be mediated mainly by the NMDA receptor that, apart from promoting cell death, due to its greater Ca^2+^ permeability, has a high affinity for glutamate and a slow inactivation [83,84]. Glutamate excitotoxicity is implicated in the mtDNA alteration or DNAoxidation–related mitochondrial dysregulation in retinal neurodegeneration [85]. Glutamate excitotoxicity over-activity leads to neuronal cell death through high levels of glutamate and the over-activation of NMDA receptors. The excitotoxic affection to RGCs may be involved by the increased glutamate synthesis or a decreased glutamate clearance [86].

## 4. WNT/β-Catenin Pathway

WNT name is derived from Wingless drosophila melanogaster and its mouse homolog Int. WNT/β-catenin pathway is involved in numerous signaling and regulating pathways, such as embryogenesis, cell proliferation, migration and polarity, apoptosis, and organogenesis [87]. However, during numerous pathological states, the WNT/β-catenin pathway can be dysregulated, such as inflammatory, metabolic and neurological disorders, tissue fibrosis and cancers [88].

The WNT pathway belongs to the family of secreted lipid-modified glycoproteins [89]. WNT ligands are secreted by both neurons and immune cells located in the central nervous system [90]. WNT pathway dysregulation contributes to several neurodegenerative diseases [91,92,93,94,95]. The WNT pathway has the main step known as the β-catenin/T-cell factor/lymphoid enhancer factor (TCF/LEF). Cytosolic accumulation of β-catenin is controlled by the destruction complex AXIN, tumor suppressor adenomatous polyposis coli (APC), and glycogen synthase kinase-3 (GSK-3β). Without WNT ligands, the destruction complex participates in hyper-phosphorylate cytoplasmic β-catenin and leads to its proteasomal degradation. However, in their presence, the WNT ligands bind to Frizzled (FZL) and LDL receptor-related protein 5/6 (LRP 5/6) interrupting the destruction complex and preventing β-catenin degradation into the proteasome. Β-catenin translocates to the nucleus where it interacts with TCF/LEF. This activates WNT target genes [96,97,98].

Glycogen synthase kinase-3β (GSK-3β) is one of the main inhibitors of the WNT/β-catenin pathway [99,100,101,102,103,104]. As an intracellular serine-threonine kinase, GSK-3β is a key negative regulator of the WNT pathway [105]. It is involved in the regulation of several kinds of pathophysiological signaling, such as cell membrane signaling, cell polarity, and inflammation [106,107,108]. GSK-3β acts by inhibiting cytoplasmic β-catenin and stabilizes it to induce its nuclear migration. Inflammation is an age-related process associated with the increase of GSK-3β activity and the decrease of the WNT/β-catenin pathways [109].

Recent studies have observed that glaucoma patients present an increased GSK-3β activity and thus its inhibition could be an interesting treatment [110,111]. GSK-3β is a serine/threonine kinase that is involved in numerous intracellular signaling pathways. Dysfunction of GSK-3β is involved in the pathogenesis of several diseases, including neuropsychiatric disorders [112]. GSK3β is known to be the major inhibitor of the canonical WNT/β-catenin pathway [103,113,114,115,116,117].

## 5. WNT/β-Catenin Pathway in Glaucoma

Recent studies have shown that the WNT/β-catenin pathway is involved in the pathophysiology of TM cells. This pathway could serve as a regulator of IOP [118]. Secreted frizzled-related protein 1 (sFRP1), a WNT inhibitor, is elevated in the glaucomatous TM. Exogenous sFRP1 involves high IOP [119,120]. In sFRP1-perfused human eyes, the level of β-catenin is decreased [119]. sFRP1 is associated with cell stiffness [120]. TM cells have multiple responses to the stimulus by different concentrations of sFRP1 [120]. It has been illustrated that sFRP1 is elevated in normal TM cells grown on substrates simulating the stiffness of the glaucomatous TM. Increased stiffness of the TM involves the aqueous humor outflow resistance and is leading to elevated IOP [120]. Moreover, the GSK3β, another WNT inhibitor, can decrease the activity of the WNT/β-catenin pathway and lead to ocular hypertension in association with sFRP1 [119]. It has been shown that there are two effects of WNT in glaucoma [118]. The glaucoma gene myocilin (MYOC) has been shown to be a regulator of WNT/β-catenin pathway [121]. Nevertheless, the damages induced by MYOC mutation on the WNT pathway remain unclear in the TM. The aqueous humor outflow resistance is damaged by the change in adhesion junctions and cell contact, and then IOP is dysregulated [118]. The WNT/β-catenin pathway is believed to be a novel interventional target for the treatment of glaucoma [122,123,124]. Several WNT target genes are expressed in the TM, and the WNT ligand WNT3a is dysregulated [118,119]. The overexpression of both sFRP1 or Dkk1 can increase IOP in perfusion-cultured human eyes and in mouse eyes [118,119]. Moreover, the cotreatment with a small-molecule WNT pathway activator can downregulate sFRP1-induced OHT in mouse eyes. The activation of WNT/β-catenin pathway in the TM using lithium chloride decreases the production of some ECM and matricellular proteins [125,126]. WNT/β-catenin signaling and K-cadherin expression are major for the control of IOP, and the downregulation of this pathway leads to IOP elevation in glaucoma [127]. Recent studies have shown that active WNT/β-catenin pathway inhibits fibrosis-associated proteins in the TM and that the POAG-associated WNT antagonist sFRP1 increases ECM deposition, TM cell stiffness [120] and IOP [118,119]. Moreover, recent findings have shown that the WNT/β-catenin can regulate TM homeostasis and IOP by a cross-inhibit circle with TGF-β signaling [126].

## 6. WNT/β-Catenin Pathway and the Altered Pathways in Glaucoma

### 6.1. WNT/β-Catenin Pathway and Oxidative Stress

FoxO (Forkhead box class O) transcription factors are the main intracellular controllers of numerous metabolic signaling such as glucose production, and the cellular response to oxidative stress [128]. ROS production is associated with the inhibition of the WNT pathway by diverting β-catenin from TCF/LEF to FoxO [129]. This leads to the accumulation and binding of β-catenin to FoxO as a cofactor, and in increasing FoxO transcriptional activity in the nucleus [130,131]. FoxO stimulates apoptotic genes [132,133,134]. FoxO3a stops the cell-cycle by stimulation of the production of the cyclin-dependent kinase inhibitor p27 kip1 and the inhibition of cyclin D1 expression [135,136]. The activation of FoxO induces apoptosis [137]. However, the activation of the WNT pathway can downregulate FoxO3a in the cytosol to prevent the loss of mitochondrial membrane permeability, cytochrome c release, Bad phosphorylation, and activation of caspases which activates ROS production and oxidative stress [138].

### 6.2. WNT/β-Catenin Pathway and Inflammation

The stimulation of the WNT pathway cascade restrains inflammation and leads to neuroprotection via interactions between microglia/macrophages and astrocytes [139,140].

Several studies have shown negative crosstalk between WNT/β-catenin pathway and NF-ϰB pathway, one of the main markers of inflammation [141]. The NF-ϰB transcription factor family belongs to five members in the cytosol under non-activated conditions: NF-ϰB 1 (p50/p105), NF-ϰB 2 (p52/p100), RelA (p65), RelB and c-Rel [142]. Β-catenin complexes with RelA and p50 to diminish the activity of the NF-ϰB signaling [143]. Moreover, by interacting with the PI3K, β-catenin inhibits the functional activity of NF-ϰB [144]. This inhibitory function of β-catenin on NF-ϰB activity has been observed in numerous cell types, such as fibroblasts, epithelial cells, hepatocytes and osteoblasts [141]. In parallel, the overactivation of GSK-3β leads to an inhibition of the β-catenin and then an activation of the NF-ϰB pathway [145]. The potential protective action of β-catenin was due to the activation of PI3K/Akt pathway and thus the reduction of TLR4-driven inflammatory response in hepatocytes [146]. NF-ϰB activation leads to the diminution of the complex β-catenin/TCF/LEF by the upregulation of LZTS2 in cancer cells [147]. DKK, a WNT inhibitor, was a target gene of the NF-ϰB pathway leading to negative feedback to diminish the β-catenin signaling [148]. Activated Β-catenin inhibits the NF-ϰB -mediated transcription of pro-inflammatory genes. This effect is controlled by the GSK-3β. GSK-3β is a direct inhibitor of the β-catenin levels and an activator of the NF-ϰB pathway [149,150].

### 6.3. WNT/β-Catenin Pathway and Glutamatergic Pathway

β-catenin activates EAAT2 and glutamine synthetase (GS) at the transcriptional level in progenitor-derived astrocytes through the activation of TCF/LEF [151]. The knockdown of β-catenin leads to the diminution of EAAT2 and GS expression in the prefrontal cortex [152]. In astrocytes, the inhibition of β-catenin is associated with diminution of both EAAT2 and GS expression [153]. The dysregulation of the WNT/β-catenin pathway induces glutamate excitotoxicity resulting in the increase of both inflammation and exudative stress [153].

## 7. Cannabidiol

Cannabinoids refer to a heterogeneous group of compounds classified into three major groups: endogenous, synthetic and phytocannabinoids [31,154]. CBD is a non-psychotomimetic phytocannabinoid derived from *Cannabis sativa plant.* The Cannabis sativa plant produces more than 66 compounds, such as delta9-tetrahydrocannabinol (THC), responsible for anxiogenic effects, and CBD, the major non-psychotomimetic compound in the plant [155]. CBD attenuates brain damage associated with neurodegeneration. Humans tolerate a high dose of CBD [156]. Moreover, CBD can interact with synaptic plasticity and induces neurogenesis. The mechanisms of the CBD effects remain unclear but have multiple pharmacological targets. Traditional medicines use Cannabis sativa for centuries. CBD, one of the main compounds of Cannabis sativa, has recently presented numerous interesting actions in many neuropsychiatric disorders [157]. CBD presents a large spectrum of possible therapeutic properties such as anxiolytic, antidepressant, neuroprotective, anti-inflammatory and immunomodulatory [31]. Cannabinoids could be considered as a new class of drugs because of their possible actions on neuropsychiatric disorders [158]. CBD has a potential therapeutic role in neuropsychiatric disorders such as schizophrenia, epilepsy, addiction and neonatal hypoxic-ischemic encephalopathy [159]. CBD can activate WNT/β-catenin and PI3K/Akt pathways and produces therapeutic effects in schizophrenia [160,161,162].

## 8. Cannabinoids in Glaucoma

CBs could have a major role in IOP control through the interaction with the ciliary muscle and Schlemm’s canal, and by the modulation of cyclooxygenase-2 (COX-2) [163]. These actions are obtained by the interaction with CB1 receptor but also by the modulation of cyclooxygenase (COX) pathway [164]. CB1 is expressed in both retina and anterior eye structures including TM, Schlemm’s canal, iris, ciliary body muscle, and ciliary pigmented epithelium. Several pathways could be implicated in the IOP lowering action of CBs by the regulation of aqueous humor production and outflow (trabecular and uveoscleral) [165]. Activation of the CB1 receptor in the ciliary muscle could also induce vasodilatation with consequent reduction of aqueous humor production [166]. Nevertheless, the exact role of CBs in the regulation of IOP remains unclear [27]. In parallel, CBs inhibit glutamate and nitric oxide release by the activation of pre-synaptic CB receptors leading to higher neuronal excitability and synaptic plasticity [28]. Glutamate pathway can regulate the RGC death through the stimulation of nitric oxide synthase and the increase in oxidative damages. Glutamate pathway in glaucoma is well investigated [27]. The anti-inflammatory actions of CBs could also have a role in neuroprotection. Stimulation of CB1 and CB2 receptors in the retina and CNS downregulates the production of nitric oxide and inflammatory cytokines which are responsible for OS and RGC death [167]. In the TM, the reduction of OS could also be obtained by ROS blockage without any CB receptor activation, such as activation of the WNT pathway [168].

Nevertheless, CBD could have an opposing effect on IOP by increasing or decreasing it [169]. The increase of IOP by CBD could be the result of the antagonist role of CBD on CB1 receptor [169]. The absence of the effect of CBD on IOP could be due to the direct and indirect activity at GPR18 receptor and CB1 receptor which could be both deleted. CBD is activated on GPR18 [170] to interrupt the activity of FAAH [171], responsible for the elevation of acylethanol-amines, such as AEA, one of the precursor of GPR18 [172]. Diurnal action of CB1 and activation of GPR18 remain unstudied. Time of day and broadly speaking pressure, which is higher during the day, regulate the pressure in the eye. Mice present a nocturnal and reversed cycle of GPR18 which participate in lowering eye pressure. Thus, diurnal signaling should have a major role in the ocular response of CBD, which is different between humans and mice [173]. Moreover, gender different effects could be involved in IOP-response to CBD, by interacting with GPR119 ligand. Female mice show lower ocular pressure under CBD administration, whereas it is not the case for male mice [174]. Furthermore, a low dose of CBD administration may have no significant IOP-lowering effect [27,175]. However, these different mechanisms remain unclear.

## 9. Activation of the Canonical WNT Pathway by Cannabidiol: A Potential Therapeutic Strategy for the Altered Pathways in Glaucoma

### 9.1. Cannabidiol and WNT Pathway

Dysfunction of GSK-3β is involved in the pathogenesis of several diseases, including neuropsychiatric disorders [112]. GSK-3β is a regulator of several pathways such as inflammation, neuronal polarity or either cell membrane signaling [107]. GSK3β is known to be the main inhibitor of the WNT/β-catenin signaling [103,113,114,117]. GSK-3β downregulates the canonical WNT/β-catenin pathway by inhibiting β-catenin cytosolic stabilization and its translocation in the nucleus [176]. Moreover, several studies have shown a link between neuro-inflammation and the increase of the GSK-3β activity and in parallel the decrease of the WNT/β-catenin pathway and the protein kinase B (Akt) pathway [99]. CBD downregulates the expression of GSK-3β through the promotion of the PI3K/Akt signaling [100,177]. PI3K/Akt signaling regulates GSK-3β activity [178]. Cannabinoids control the PI3K/Akt/GSK-3β axis [179,180]. Genes encoding for the PI3K/Akt pathway is increased in CBD-GMSCs (mesenchymal stem cells derived from gingiva treated by CBD) [100].

### 9.2. Cannabidiol and Oxidative Stress

Energy and glucose metabolisms involved during oxidative stress are mainly controlled by the intracellular FOXO transcription factors (FOXO1, 3a, 4) [128]. The interaction between β-catenin and FOXO transcription factors promotes cell quiescence and cell cycle arrest. Β-catenin blocks its transcriptional complex with TCF/LEF through the interaction with FOXO-induced ROS [129]. Β-catenin does not translocate to the nucleus and thus accumulates in the cytosol to inactivate the WNT/β-catenin pathway [130,131].

CBD can reduce the redox balance through the modification of both the level and activity of oxidants and antioxidants [181]. CBD stops the free radical chain reactions through the capture of free radicals and then by reducing their activities [182]. CBD downregulates the oxidative conditions through the prevention of the formation of superoxide radicals, generated by xanthine oxidase (XO), NADPH oxidase (NOX1 and NOX4) [183,184]. Moreover, CBD can enhance the diminution in NO levels in the liver of doxorubicin-treated mice [185]. CBD diminishes reactive oxygen species (ROS) production through the chelation of transition metal ions implicated in the Fenton reaction to form extremely reactive hydroxyl radicals [186]. CBD acts on the classic antioxidant butylated hydroxytoluene (BHT) to prevent the dihydrorodamine oxidation in the Fenton reaction [187].

The antioxidant activity of CBD is characterized by the activation of the redox-sensitive transcription factor which refers to the nuclear reythroid 2-related factor (Nrf2) [188] responsible for the transcription of cytoprotective genes [189]. Superoxide dismutase (SOD) and enzymatic activities of Cu, Zn and Mn-SOD, which are responsible for the metabolism of superoxide radicals, are increased by CBD [190]. Glutathione peroxidase and reductase are increased by CBD and decrease the malonaldehyde (MDA) levels [191]. Enzymatic activities are altered during oxidative modifications of proteins. CBD, by targeting glutathione and cytochrome P450, car inhibit their biological activity to decrease oxidative stress [185,192]. Moreover, through the diminution of ROS levels, CBD can prevent and protect non-enzymatic antioxidants [190], including vitamins A, E and C [193].

### 9.3. Cannabidiol and Inflammation

Cannabinoids present anti-inflammatory action by endogenous receptors, such as cannabinoid receptor 1 (CB1) and cannabinoid receptor 2 (CB2) [194]. N-Oleoyl glycine (OLGly), a lipoamino acid, increases adipogenic genes including PPARγ, a marker of inflammation, and the mRNA expression of CB1 receptor. The inhibition of CB1 receptor by its antagonist SR141716 downregulates the actions of OLGly on the expression of PPARγ. Moreover, OLGly activates the Akt pathway and inhibits FoxO activity [195]. CBD can bind PPARγ [162,196]. PPARγ is a main factor of inflammation by interacting with NF-κB. This bind occurs between the ligand-binding domain of PPARγ and the Rel homology domain region of the p65 subunit of NF-κB. Proteasomal degradation of p65 is caused by Lys48-linked polyubiquitin of the ligand-binding domain of PPARγ [197]. Thus, PPARγ can modulate inflammation through the ubiquitination proteasomal degradation of p65 leading to the control of cyclooxygenase (COX2), TNF-α, IL-1β and IL-6 [162]. PPARs are ligand-activated transcription factors that bind PPRE (PPAR-response elements). PPARs are implicated in numerous pathophysiological mechanisms, such as cell differentiation, protein metabolism, lipid metabolism, carcinogenesis [198,199], adipocyte differentiation, insulin sensitivity and inflammation [200,201]. PPARγ ligands, such as thiazolidinediones (TZDs), are able to decrease inflammatory activity [202]. Negative crosstalk has been well described between PPARγ and the WNT pathway [113,203,204,205]. The PI3K/Akt pathway, which is positively induced by β-catenin [117,204,206,207,208], acts through the phosphorylation of GSK-3β to negatively control the PPARγ expression [209]. PPARγ agonists decrease β-catenin expression by overactivating GSK-3β [210]. Moreover, PPARγ agonists stimulate Dickkopf-1 (DKK1) activity to diminish the canonical WNT/β-catenin pathway and then downregulate the differentiation of fibroblasts [211]. Moreover, PPARγ agonists stimulate GSK-3β to diminish β-catenin expression [210]. In parallel, β-catenin directly inhibits NF-κB activity [149,150].

### 9.4. Cannabidiol and Glutamatergic Pathway

Few studies have investigated the interaction between the endogenous cannabinoid system and the glutamatergic pathway in the brain [212]. CBD diminishes the glutamate release in neural signaling implicated in compulsive behavior [213]. Many studies highlighted that the actions of CBD on dopamine and GABA levels were correlated with its strong anti-oxidant properties through the modulation of nitric oxide synthase expression and the inhibition of ROS-generating NADPH oxidases [214]. However, endogenous cannabinoids can bind to the cannabinoid CB1 receptor and dampen presynaptic glutamate release [215]. Moreover, the inhibition of GSK-3β can decrease EAAT3 activity [82]. Nevertheless, the relation between CBD and the glutamatergic pathway remains unclear. CBD can block the actions of CB1R/CB2 combined receptor agonist [216] and can act as a CB1R antagonist [217].

## 10. Conclusions

Currently, even if CBs are well documented in the literature, few investigations have studied CBD as a possible alternative therapeutic way to treat glaucoma patients. Nevertheless, CBD could appear to be interesting in glaucoma by targeting both oxidative stress, inflammation and the glutamatergic pathway through the activation of the WNT/β-catenin pathway. The action of CBD is mainly involved by its negative interaction with GSK-3β, the main inhibitor of the WNT/β-catenin pathway. In glaucoma, the WNT/β-catenin is downregulated to allow the stimulation of oxidative stress, inflammation and glutamatergic pathway. Future prospective studies should focus on CBD and its different actions in glaucoma.

## Data Availability

Not applicable.

## Abbreviations

GSK-3β	Glycogen synthase kinase-3β
LRP 5/6	Low-density lipoprotein receptor-related protein 5/6
NF-ϰB	nuclear factor ϰB
PPARγ	Peroxisome proliferator-activated receptor gamma
PI3K-Akt	Phosphatidylinositol 3-kinase-protein kinase B;
TCF/LEF	T-cell factor/lymphoid enhancer factor;
TNF-α	tumor necrosis factor alpha.
MDPI	Multidisciplinary Digital Publishing Institute
DOAJ	Directory of open access journals
TLA	Three letter acronym
LD:	linear dichroism
